# The Immunogenetic Conundrum of Preeclampsia

**DOI:** 10.3389/fimmu.2018.02630

**Published:** 2018-11-13

**Authors:** A. Inkeri Lokki, Jenni K. Heikkinen-Eloranta, Hannele Laivuori

**Affiliations:** ^1^Research Programs Unit, Immunobiology Research Program, University of Helsinki, Helsinki, Finland; ^2^Bacteriology and Immunology, University of Helsinki and Helsinki University Hospital, Helsinki, Finland; ^3^Obstetrics and Gynecology, University of Helsinki and Helsinki University Hospital, Helsinki, Finland; ^4^Medical and Clinical genetics, University of Helsinki and Helsinki University Hospital, Helsinki, Finland; ^5^Institute for Molecular Medicine Finland, Helsinki Institute of Life Science, University of Helsinki, Helsinki, Finland; ^6^Faculty of Medicine and Life Sciences, University of Tampere, Tampere, Finland; ^7^Department of Obstetrics and Gynecology, Tampere University Hospital, Tampere, Finland

**Keywords:** preeclampsia, genetics, complement, major histocompatibility complex, FLT1, autoimmunity, pregnancy

## Abstract

Pregnancy is an immunological challenge to the mother. The fetal tissues including the placenta must be protected from activation of the maternal immune system. On the other hand, the placental tissue sheds into the maternal circulation and must be adequately identified and phagocytized by the maternal immune system. During a healthy pregnancy, numerous immunosuppressive processes take place that allow the allograft fetus to thrive under exposure to humoral and cellular components of the maternal immune system. Breakdown of immune tolerance may result in sterile inflammation and cause adverse pregnancy outcomes such as preeclampsia, a vascular disease of the pregnancy with unpredictable course and symptoms from several organs. Immunological incompatibility between mother and fetus is strongly indicated in preeclampsia. Recently, genetic factors linking immunological pathways to predisposition to preeclampsia have been identified. In this mini-review genetic variation in immunological factors are discussed in the context of preeclampsia. Specifically, we explore immunogenetic and immunomodulary mechanisms contributing to loss of tolerance, inflammation, and autoimmunity in preeclampsia.

## Introduction

Preeclampsia is a heterogeneous vascular disease of the human pregnancy that presents in a previously normotensive woman during the second half of the pregnancy with hypertension and proteinuria, or preeclampsia-associated signs in the absence of proteinuria (1, 2). Preeclampsia occurs in 3% of pregnancies (3), and it is one of the most important causes of maternal and fetal morbidity and mortality worldwide. The etiology of preeclampsia is incompletely understood, but it has its origins in early pregnancy and delivery of the placenta is the only cure (4). Two distinct subtypes has been frequently used in the literature based on the timing of the disease onset/delivery: early-onset <34 + 0 and late-onset ≥34 weeks of gestation. However, better understanding of the etiology and different subtypes is needed for early recognition and preventive measures.

Preeclampsia is considered a two stage-disease in which poorly perfused placenta produces factor(s) leading to systemic vascular disease and the clinical manifestations of preeclampsia (5).

**Figure 1:**
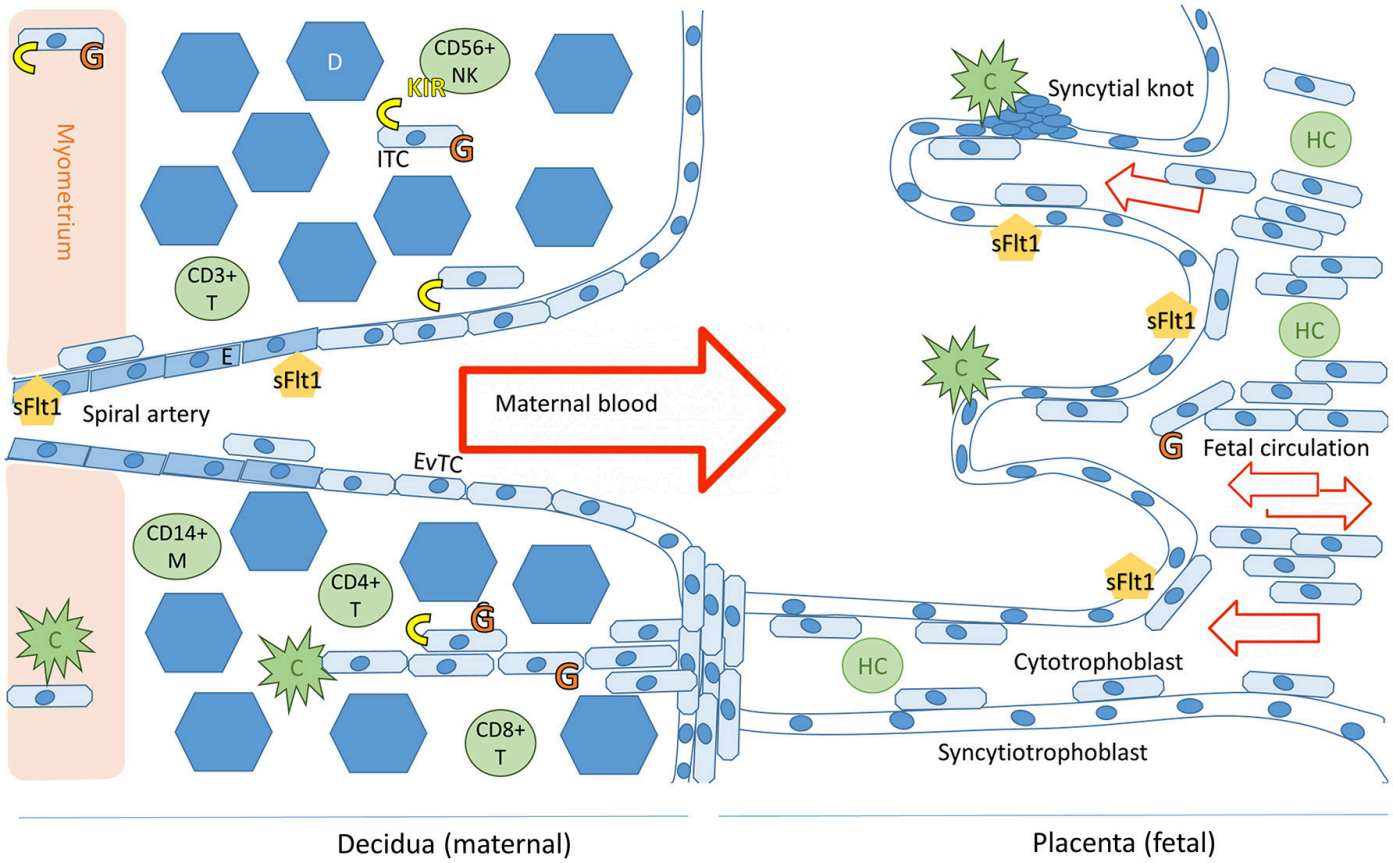
Schematic representation of the maternal-fetal interface and its immunologicalplayers. Trophoblast cells (fetal) and endothelial cells (maternal) express sFlt1. The placental villi are shown in the right side of the image and decidua on the left. Invading trophoblast cells will encounter maternal complement system (C) in the decidua and in the intervillous space. Invading extravillous trophoblasts express HLA-C and HLA-G receptors (in yellow and orange, respectively), expression of HLA-G by villous trophoblast cells in the placenta decreases during the course of the pregnancy (7). Successful trophoblast invasion will extend to the vascular layers of myometrium and invade the uterine spiral arteries, where endovascular trophoblast cells (EvTC) will replace endothelial cells (E) causing remodulation and relaxation of the spiral artery to allow for non-turbulent high volume low pressure circulation into the intervillous space. Interstitial trophoblast cells (ITC) will remain in the maternal tissue creating tolerance of the fetal tissue in the maternal immune system. Hofbauer cells (HC) are the predominant immune cell population in the villi throughout placental development. The decidual immune cell population consists of macrophages (M), natural killer cells (NK), and populations of T-cells (T). Tolerance inducing Treg and Breg cells in particular are essential for a healthy pregnancy (8). Figure adapted from Lokki 2017 PhD thesis (9).

**Figure 2:**
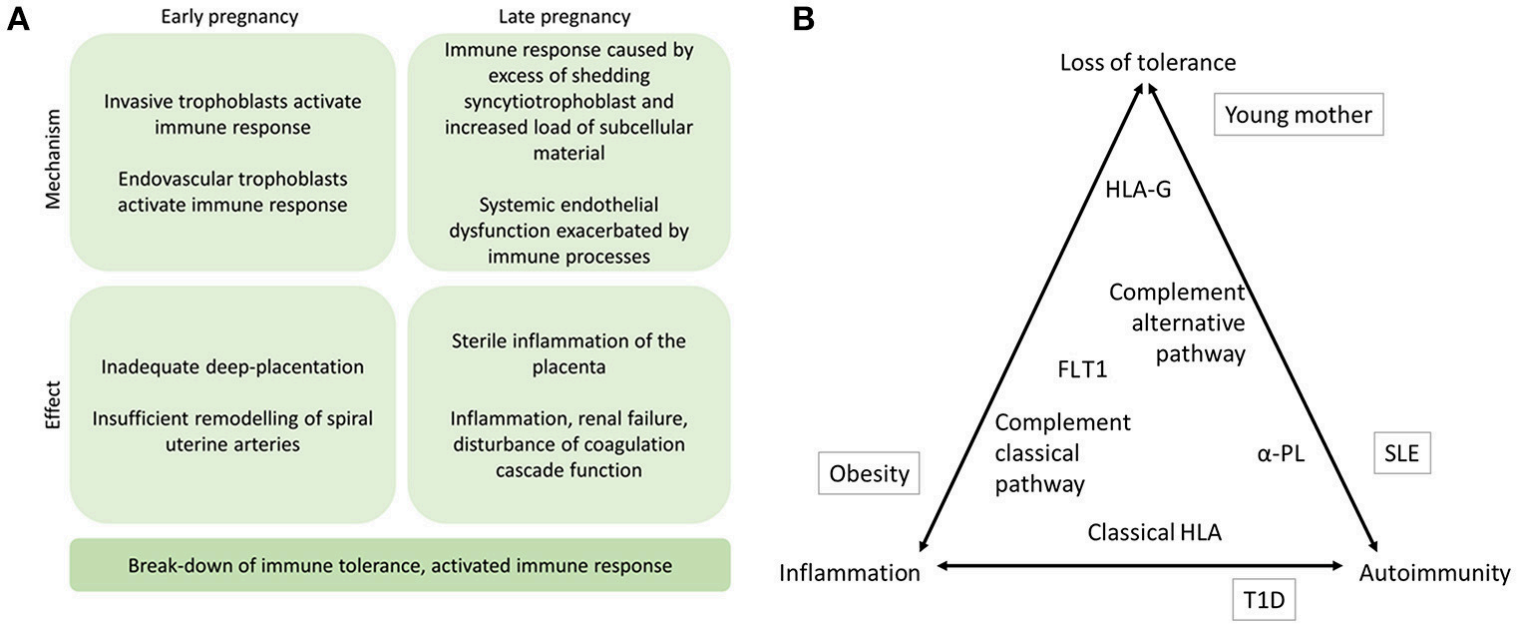
**(A)** The proposed mechanisms of immune response in the etiology of preeclampsia. Immune mechanisms contribute to preeclampsia in early and late stages of the pregnancy. Specifically, if alternative pathway (AP) of complement system fails to recognize the invading trophoblast cells at the placental bed or in the uterine arteries, placentation may remain superficial and maternal uterine spiral artery remodeling may be compromised. In later stages of the pregnancy, complement activation aiming to facilitate phagosytosis of excess syncytiotrophoblast debris may induce sterile inflammation of the placenta locally. Concurrently, systemic endothelial dysfunction including disturbed coagulation function may be aggravated by immune activation and result in inflammation and shifting of Th2 to Th1 helper cell as well as disturbance of Treg and Th17 cell balances thereby further contributing to activation of the maternal immune response including erroneous antigen presenting in patients with applicable HLA alleles (11). **(B)** The triad of immune mechanisms in preeclampsia. The placement of contributing factors represents their suggested role in contributing to loss of tolerance, inflammation, and autoimmunity. Environmental risk factors outside in boxes, and the immunogenetic factors discussed in this review are inside the triangle. α-PL, anti-phospholipid antibodies; HLA, human leukocyte antigen; FLT1, Fms-like tyrosine kinase 1; T1D, type 1 diabetes.

At 8 weeks of gestation, the trophoblast cells invade from the placenta into the maternal tissue and into the uterine arteries. These endovascular trophoblast cells facilitate the remodeling of spiral uterine arteries, which is essential for a healthy pregnancy. In order for the placentation process to be sufficient to support a healthy pregnancy, the extravillous trophoblast cells must avoid detection by the alternative pathway, subsequent complement activation, and immune response ((4, 6); Figure 1). Immunogenetic susceptibility to preeclampsia may have effect in the early stages of pregnancy whereby through loss of maternal tolerance toward the fetal components, the process of placentation is impaired. On the other hand, during the third trimester, underlying immunogenetic predisposition may aggravate sterile inflammation, which is exacerbated by systemic endothelial dysfunction in the mother's vasculature and result in progression of preeclampsia ((10) Figure 2A).

Data from epidemiological studies suggest that maternal and paternal genes through fetus affect the risk of preeclampsia, and its genetic basis is polygenic. Heritability of preeclampsia has estimated 55% with greater maternal (35%) than fetal (20%) contribution (12). Genes coding for components of the immune system are among the more important candidates in the quest to pinpoint clinically relevant genetic association. This is also evidenced by numerous non-genetic studies and observations involving components of the immune system (13–15). Activation of alternative pathway of complement activation has been shown to coincide with the critical weeks of placentation (16, 17). The role of decidual monocyte populations in healthy and pathological pregnancies have been reviewed elsewhere (18).

Genetic risk profile for preeclampsia is currently poorly characterized. The genetic studies have suffered from non-reproducibility and been lacking functional validation. Recently, in a large genome wide association study of preeclampsia a first robust association in the fetal genome was found in the common variant near Fms related tyrosine kinase gene (*FLT1*) encoding anti-angiogenic factor Fms-like tyrosine kinase 1 (FLT1) (19). Our group has published protective maternal low-frequency variants in the same gene (20).

In this mini-review we explore the immunogenetic role of *FLT1* in preeclampsia and selected genetic studies implicating loss of immune tolerance in early pregnancy or late pregnancy inflammation in preeclampsia. Dysregulation of complement system and autoimmunity are discussed in detail as potential causes of loss of maternal tolerance, while obesity is considered a possible cause of inflammation.

## Immunogenic FLT1? an evolutionary perspective

The anti-angiogenic factor, soluble FLT1 is also known to have an anti-inflammatory function (21). FLT1 is expressed on inflammatory cells in addition to endothelial and trophoblast cells (22). In areas of Africa, where *Plasmodium falciparum* malaria is endemic, first pregnancies share a particular risk of not only preeclampsia but also of placental malaria (23). In placental malaria, the fetal tissue will express an excess of sFLT1 apparently in an attempt to regulate the maternal inflammatory response thereby reducing the rate of spontaneous abortions (24). Consequently, positive selection on a genetic variant with capacity to resist placental malaria by increasing sFLT concentration may have influenced *FLT1* allele frequencies within the general population enough to introduce a novel risk to preeclampsia (25).

Soluble FLT1 is conserved across vertebrates. The human FLT1 protein contains two tyrosine kinase catalytic (TyrKc) domains, three domains from the immunoglobulin (Ig) cell adhesion molecule (cam) subfamily (Igcam), one Ig-like domain, and one true Ig domain (26). In a detailed molecular evolutionary analysis, in contrast to other related proteins, in FLT1, only the TyrKc domains located at amino acids 819-933 and 991-1157 were found to be conserved across vertebrates (26). Large degree of variance between related proteins may be a reflection of recent evolutionary selection pressure on the FLT1. Malaria is known to be a potent source of immunological selection. Together this evidence is in support of possible thus far poorly understood immunological roles of the FLT1.

The major contributor to sFLT1 load in human pregnancy is the recently evolved isoform sFLT1-e15 (27). Overexpression of the primate specific isoform sFLT1-e15a is also associated with preeclampsia suggesting, that this novel isoform harbors thus far unexplained fitness advantages (27, 28). Assuming that sFLT1 is pathogenic, it is thereby possible that in non-primate mammals' conditions that lead to pregnancy-associated pathological rise in sFLT1 do not exist. On the other hand, it is also possible that the sFLT1 in humans has evolved specific functions, patterns of expression, or regulatory mechanisms that are essential for development of preeclampsia (25).

Further evidence of the immunological interactions of FLT1 is derived from a murine model, where increase in complement activation resulted in increased levels of FLT1 (29). Monocytes can be stimulated to express an excess of FLT1 when exposed to complement activation products C3a and C5a *in vitro* (29). Additionally, nuclear factor of activated T-cells (NFAT) transcription factors are involved in expression of mRNA of inflammatory cytokines, sFLT1-e15, and FLT1, as well as, and secretion of sFLT1 from primary human cytotrophoblasts (30). NFAT transcription factors may in further studies prove to be another link between FLT1 and immune response. Furthermore, angiogenic dysregulation may play a role in activation of the classical pathway in the kidney in a murine model of preeclampsia as evidenced by C4 deposition in the tissue in presence of excess sFLT1 (31).

*FLT1* 3'UTR dinucleotide repeat polymorphism have been shown to influence the expression of FLT1 and fetal outcome in the context of placental malaria with possible immunomodulatory effect (23). As far as we know, the distribution of these repeat polymorphisms in preeclampsia has not been explored.

## Tolerating offspring: dual role of complement system in recognizing and clearing of fetal material

Complement system is an ancient part of innate immunity, which consists of cell surface-bound and freely circulating proteins that interact in a cascade of activation and regulation. Complement system has the capacity to discriminate between self- and non-self-cells and particles, and thereby maintain tolerance, or activate adaptive immunity. Complement activation can lead to inflammation, cell death, and tissue destruction. However, complement system also has a crucial role as a facilitator of phagocytosis thereby clearing debris and altered cells, in addition to removing pathogens. To protect own tissues from complement-mediated destruction and death, pathways of complement activation must be carefully regulated. Complement system has been studied extensively in preeclampsia, but genetic association studies linking components of the complement system to preeclampsia are not as plentiful.

Preeclampsia has previously been likened to thrombotic microangiopathies (TMA), which are caused by inadequate regulation of the complement system. In TMA, complement attacks against endogenous tissue structures such as endothelial cells and blood cells causing vascular damage and kidney failure. Pregnancy can act as a trigger of TMA syndromes. Atypical hemolytic uremic syndrome (aHUS) is a complement disease of the kidney with a TMA mechanism (32). Dysregulation of alternative pathway of complement system is indicated in aHUS (33). Similarly, most preeclampsia associations in complement system are found in the alternative pathway (34, 35).

The component C3 is in the core of the complement system. It can be activated by three different pathways. C3 can also become spontaneously activated in the human serum (32). Via the alternative pathway of complement activation, C3 is spontaneously activated and cleaved into activation products C3a and C3b in the absence of complement regulators. According to the sequence context, a haplotype spanning the active domains of C3 may predispose or protect from severe preeclampsia in a Finnish population (36, 37). We found a haplotype of 16 SNPs spanning the functionally critical sections in the middle of the gene. In this haplotype, three SNPs have most robust independent associations to severe preeclampsia further supporting its functional significance (36).

The results of this and other studies indicate that parallel to mice, C3 also plays a central role in the healthy human pregnancy (38, 39). The mechanism of haplotype association to severe preeclampsia is unclear but the effect may be due to functional or regulatory attributes of this region. For example, functional effect may affect the extravillous trophoblasts' capacity to evade complement activation by C3 binding, thereby compromising deep placentation and spiral artery remodeling in early pregnancy. Inflammation caused by excess complement activation may also be involved in a later stage of preeclampsia influencing severity of symptoms such as hypertension (40).

Immune complexes or opsonisation of the target surface by C1 complex triggers the activation of the classical pathway of complement system. Clearance of placenta-derived particles is crucial when preeclampsia symptoms develop in later pregnancy. Classical pathway activation results in cleavage of C4, which is a homolog of C3. C4 is present in two proteins, C4A and C4B, which are coded by usually two copies of each gene. While copy number variation of both *C4* is common, zero copies of both resulting in complete *C4* deficiency are very rare (41). Results of a pilot study conducted on mother-infant pairs with early-onset (delivery < 34 weeks of gestation) or late-onset (delivery ≥ 34 weeks of gestation) preeclampsia and non-preeclamptic controls suggest that deficiencies in *C4* may predispose to preeclampsia (14). *C4A* or *C4B* deficiencies were found almost twice as often in women with early-onset preeclampsia than in healthy controls. *C4A* deficiencies are observed in 16% of general population in Finland (42). In preeclampsia, *C4A* deficiencies were found in 40% (2/5) of women with late-onset preeclampsia and in 43% (3/7) of women with early-onset preeclampsia. None were observed in controls (*n* = 7). The copy number of *C4* seems to decrease with the severity of preeclamptic symptoms (14). *C4A* deficiencies have previously been linked to autoimmune diseases (43). The patients in the preeclampsia study did not suffer from autoimmune diseases. The high incidence of *C4A* deficiency in preeclampsia supports the importance of classical pathway of complement system in preeclampsia.

Membrane co-factor protein (MCP, CD46) has the capacity to regulate both alternative and classical pathways of complement activation by binding to C3b or C4b and acting as a co-factor to the inactivator enzyme Factor I. MCP is a widely expressed type 1 membrane bound protein. aHUS, can be caused by mutations in *MCP* (44–47). Proteinuria is one of the cornerstone symptoms of preeclampsia, but its degree varies between patients. Renal dysfunction due to uncontrolled complement activation has been suspected to be the underlying link between preeclampsia and kidney diseases. To investigate whether sequence variants in the *CD46* might predispose to preeclampsia, we sequenced the *MCP* gene in preeclamptic women with severe proteinuria and in non-preeclamptic controls (37, 48). The results of this study do not corroborate the previously reported association of A304V to severe preeclampsia in a cohort of autoimmune pregnancies (49). We observed similar minor allele frequency (MAF) of ~6% in cases and controls. We also found one control woman, who was homozygous to 304^*^V allele. Heterozygosity for another functional single nucleotide polymorphism (SNP) K32N (rs150429980) was found in one preeclamptic woman and one control. Thereby results are inconclusive. It is possible, however, that MCP plays a part in a particular subtype of preeclampsia, due to the heterogenous nature of the disease.

The complement co-factor I (coded by *CFI*) is a serine protease that inactivates C3b and C4b in the presence of a co-factor protein, such as complement factor H (FH) and MCP. In the PROMISSE cohort consisting of patients with anti-phospholipid antibodies or systemic lupus erythematosus (SLE), two severe preeclamptic patients with a history of complicated pregnancies were found to carry the loss-of-function mutation I398L in *CFI* (49). Furthermore, a mutation in the *CFH* the gene coding for FH with unknown functional consequence was found in another patient (49). While FH is mainly effective in inhibition of the alternative pathway, MCP and factor I have the capacity to regulate both alternative and classical pathways of complement activation.

## Immunogenetic predisposition for compromised tolerance

Major histocompatibility (MHC) in chromosome 6 (6p21.3), is the most polymorphic region of the human genome as a result of diverse and shifting immunological selection pressures. Many of the genes in the MHC code for proteins with immunological function. Genes coding for C4A and C4B are located in the MHC as are the genes coding for human leukocyte antigen (HLA) receptors.

At least two autoimmune diseases exist that harbor an increased susceptibility to preeclampsia. Among other immunological defects, aberrant NK cell biology has also been implicated in both, SLE and Type 1 Diabetes. Together these observations might shed light to the disease mechanisms in pregnancy complications.

The proportion of natural killer (NK) cells increases markedly in the uterus/endometrium during implantation and they likely have an important function during early stages of placentation (50, 51). Accordingly, genotypes of KIR receptors on the NK cells in combination with genotypes of their ligands, HLA-C on fetal trophoblast cells have been under investigation in preeclampsia (52–54).

SLE is characterized by a diminished number of NK cells with variety of functional abnormalities (55). SLE shares many symptoms with preeclampsia, including hypertension, proteinuria, and thrombocytopenia. SLE carries a 2- to 4-fold increase in risk of preeclampsia during pregnancy. In a Swedish population-based registry study, the risk for severe preeclampsia in SLE patients was 4.3% (56). Among the MHC loci, the HLA-DRB1^*^15:01, one of the strongest susceptibility loci for SLE in European-descent populations, is also associated to reproductive failure, i.e., recurrent pregnancy loss and secondary recurrent pregnancy loss (57, 58).

Among pregnant women with Type 1 diabetes (T1D), 15–20% develop preeclampsia (59), and nephropathy further increases the risk of preeclampsia to up to 42–52% (60), which raises questions of shared pathologies. NK cells have a crucial role in trophoblast invasion and spiral artery remodeling in the early stages of pregnancy, as well as in the recognition of the allograft fetal cells. Diverse aberrations of NK cell function are widely evidenced in T1D [reviewed in (61)]. It has been shown, that during the diabetic pregnancy, NK cells adhering to normal decidual endothelium are diminished in comparison to the non-diabetic control pregnancies suggested reduced number of NK cells homing to decidua in the diabetic pregnancy (62). Furthermore, the peripheral blood CD56bright NK cells from pregnant T1D patients expressed very low levels of selectin L (SELL) and alpha 4 integrin (ITGA4), which are important receptors for homing to the uterus (63). CXCL10 and CXCL12 chemokines are produced by the decidua. Their receptors CXCR3 and CXCR4, respectively, were expressed in lower levels on NK cells from T1D patients (63). Furthermore, the expression of activating receptor CD335 in the NK cell is increased during pregnancy in T1D patients. Aberrant NK cell function may result in the increased Th1/Th2 ratio and enhanced activation of intermediate and non-classical monocytes (64), both of which have been observed in preeclampsia, as well as T1D. This may contribute to the underlying mechanism of higher incidence of preeclampsia in T1D patients.

While the villous trophoblasts in the placenta are HLA null, the invasive extravillous trophoblasts (EVT) express genes belonging to the MHC, namely HLA-E, -F, -G, and -C genes (Figure 1). Trophoblast cells do not express HLA class II on the placental surface but syncytiotrophoblast, the outer most layer of placental villi, contains intracellular HLA class II antigens (65). Therefore, compatibility of maternal and fetal HLA genotypes may also influence the immune response in late pregnancy when fetal components are released from the disintegrating placenta. In this context, HLA-A, -B, -DR, and -DQ gene groups may also be relevant in preeclampsia, but thus far, this hypothesis has received little attention.

HLA-G is considered to be protective and tolerogenic during pregnancy. HLA-G is present in semen, which suggests that immune tolerance induction starts already before conception. There are several studies suggesting low or reduced levels of sHLA-G in preeclampsia patients and reduced levels of HLA-G mRNA has been observed in placentas of preeclamptic women (66–69). In a study of genetic polymorphisms, a 14-bp ins/del polymorphism in the 3'UTR of exon 8 of the HLA-G gene was associated with mRNA stability and overall HLA-G production (70). The role of HLA-G polymorphism in preeclampsia is still unresolved.

## Antiangiogenic sFLT1 and increased inflammatory response in established preeclampsia

Inappropriate maternal immune responses to trophoblast in early pregnancy may lead to abnormal placentation and set the stage for clinical preeclampsia later in pregnancy. Established preeclampsia is characterized by endothelial dysfunction and systemic inflammatory response to placental oxidative stress [Figure 2A; (71)].

It has been suggested that antiangiogenic sFLT1 sensitizes endothelial cells to pro-inflammatory factors (72). The first genome wide association study of offspring from preeclamptic pregnancies reported that common variants near *FLT1* on chromosome 13 were associated with preeclampsia (19). Incidence of preeclampsia is known to be increased in pregnancies with fetal Trisomy 13 (73) suggesting that increased placental production of sFLT1 has a role in preeclampsia susceptibility. Furthermore, the importance of the *FLT1* in the disease is evidenced by the recently discovered protective variants in the gene (20).

Normal third-trimester pregnancy is characterized by activation of peripheral blood leukocytes, which is further increased in preeclampsia (74). Increase in the maternal circulating levels of proinflammatory cytokines tumor necrosis factor alpha (TNF α), interleukin (IL)-6, and also the anti-inflammatory cytokine IL-10 in the third trimester of pregnancy in women affected by preeclampsia have been demonstrated in a meta-analysis (75).

Obesity increases the risk of preeclampsia 2- to 3-fold (76–78), but the underlying mechanisms are not fully understood. Obesity is a state of uncontrolled inflammatory responses leading to systemic low-grade inflammation and increased insulin resistance (79). Even modestly overweight women have vascular endothelial dysfunction assessed by brachial artery ultrasound flow-mediated vasodilation (80). The secretion of pro-inflammatory cytokines including TNFα and IL-6 is increased in hypertrophic adipocytes (79). Herse and coworkers have also shown that TNFα decreased sFLT1 expression in mature adipocytes (81). We and others have found lower concentration of sFLT1 in obese compared to normal-weight preeclamptic women, but not in respective normotensive pregnant women (82, 83). Associations between maternal body mass index and proinflammatory cytokines TNFα and MCP-1 in maternal plasma have been demonstrated (84). Thus, the production of sFLT1 and proinflammatory cytokines by placenta and extraplacental sources may be different in obese and normal-weight women during pregnancy and in normal and complicated pregnancies.

## Conclusion

The mechanisms regulating the immune response are central in normal pregnancy and the development of preeclampsia (Figure 2A). Mechanisms of autoimmunity, loss of tolerance, and inflammation in preeclampsia are evidenced in this mini-review (Figure 2B). Angiogenic proteins, continuous subclinical inflammation, and insulin resistance in preeclamptic women have been suggested to result in increased cardiovascular risk that with additional risk factors may result in cardiovascular disease (85–87). Thus far, investigation into the genetic background of the immunological pathogenesis of preeclampsia has mostly concentrated on the genes coding complement components and MHC. While both pathways are relevant to the early pregnancy and later clinical manifestations of preeclampsia, studies addressing other immunological mechanisms will be a welcome contribution to increase our understanding of the complex immunological interactions in the disease.

## Author contributions

All authors listed have made a substantial, direct and intellectual contribution to the work, and approved it for publication.

### Conflict of interest statement

The authors declare that the research was conducted in the absence of any commercial or financial relationships that could be construed as a potential conflict of interest.
